# Case Report: Propranolol increases the therapeutic response to temozolomide in a patient with metastatic paraganglioma

**DOI:** 10.12688/f1000research.13185.1

**Published:** 2017-12-04

**Authors:** Miguel-Angel Díaz-Castellanos, Karina Villar Gómez de las Heras, Tamara Díaz-Redondo, Encarnación González-Flores, Virginia Albiñana, Luisa-María Botella

**Affiliations:** 1 PHEiPAS, Castellón de la Plana, 12006, Spain; 2 Alianza VHL, Centro Cívico Rogelio Soto, Sabadell, 08204, Spain; 3 Sanidad Castilla-La Mancha, Toledo, Spain; 4 Servicio de Oncología, Hospital Universitario Carlos Haya, Málaga, 29010, Spain; 5 Servicio de Oncología, Hospital Universitario Virgen de las Nieves, Granada, 18014, Spain; 6 Centro de Investigaciones Biológicas, U-707 CIBERER, Madrid, Spain

**Keywords:** Paraganglioma, rare oncologic diseases, repurposing drugs, pseudohypoxic cancer syndromes

## Abstract

This case report presents the clinical evolution and management of a patient with a hereditary paraganglioma syndrome. This disease is characterized by rare tumors of neural crest origin that are symmetrically distributed along the paravertebral axis from the base of the skull and neck to the pelvis. In addition, these patients may develop renal cancer, gastrointestinal stromal tumors, pituitary adenomas, and bone metastasis in some cases. To date no successful therapeutic treatment has been reported. Total resection with postoperative radiotherapy and chemotherapy have been advocated, especially for the multiple metastasis. Here we show how the combination of high doses of the beta blocker propranolol (3 mg/Kg/day) and the DNA intercalating agent, temozolomide, has been successful in the treatment of a SDHA metastatic paraganglioma.

## Introduction

Hereditary paraganglioma and pheochromocytoma syndromes (HPP) are characterized by rare tumors called pheochromocytomas (PHEOs) and paragangliomas (PGLs). These are highly vascularized catecholamine-secreting tumors of neural crest origin (chromaffin cells derived from the adrenal medulla – pheochromocytoma – or extra-adrenal paraganglia - paragangliomas), distributed symmetrically along the paravertebral axis. Although tumors are usually histologically benign, most of them hyper-secrete catecholamines, causing high cardiovascular morbidity and mortality ^1^, and symptoms related to mass effect. If left untreated, a subset of these tumors will metastasize in bones, lungs, liver or lymph nodes ^2^. These patients can develop other rare tumors too, such as renal cancer and gastrointestinal stromal tumors. However, tumorigenesis is rare in the first decade of life.

HPP syndromes are due to pathogenic variants of genes, SDHx genes, a group of multiple nuclear genes encoding subunits of the succinate dehydrogenase (SDH) enzyme complex. Similarly to other autosomal-dominant hereditary cancer disease - von Hippel-Lindau (VHL) -paraganglioma–pheochromocytoma (PGL/PCC) syndrome is related to the hypoxia pathway ^3^. Hypoxia-inducible factor 1 (HIF1) regulates the cellular response according to oxygen levels. HIF is a heterodimer of a HIF1-α subunit, regulated by oxygenation and a β subunit. Under normal oxygen conditions, HIF1-α degradation is promoted by the E3 ubiquitin ligase pVHL (von Hippel Lindau factor). In lower oxygen cell environments, VHL recognition of HIF-1α is decreased, allowing HIF1 to promote cellular survival and growth ^4^. In cancer, the hypoxia pathway is switched by the tumoral cells leading to the tumor growth ^5^. Pseudo-hypoxic states are those leading to the hypoxia-pathway gene expression, under normoxic conditions.

Germline mutations in the onco-suppressor gene, called *Vhl* leads to the autosomal-dominant hereditary cancer disease VHL. VHL disease is clinically characterized by retinal and CNS hemangioblastomas, RCC (renal clear cell carcinoma), PCC, neuroendocrine pancreatic tumors and pancreatic cystadenomas, endolymphatic sac tumors and broad ligament cystadenomas. Closely related to this disease are the mutations involving the succinate dehydrogenase genes (SDHx genes) ^6^. The lack of function of SDHx genes leads to an inhibition of prolyl hydroxylases by alpha-ketoglutarate. These hydroxylases “target” HIF1/2-α by specific hydroxylation to be recognized and bound by VHL. In this way, hydroxylases cannot help in the degradation of HIF, and a state of pseudo-hypoxia ^7^ is created. Thus, pseudo-hypoxic states display similar hypoxia-pathway triggered gene expression, but under normoxic conditions.

Surveillance recommendations for both diseases (VHL disease and PHEO/PGL) ^2^ are very similar. It is important to suspect, localize and resect these tumors.

The present case presents the clinical evolution and management of a patient with a hereditary paraganglioma syndrome. The combination of high doses of the beta blocker propranolol (3 mg/Kg/day) and the DNA intercalating agent, temozolomide, was been successful in the treatment of the SDHA metastatic paraganglioma.

## Case description

**Figure 1.**
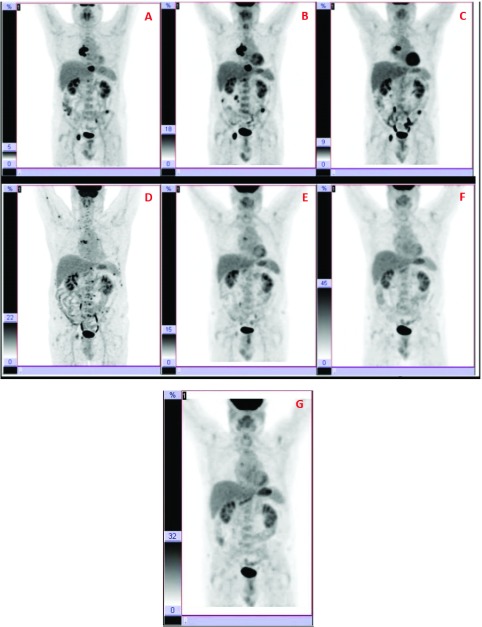
MRI and 18F-DG-PET scan. ( **A**) In October 2014, several metastasis at D6–D7, right acetabulum (3cm); vertebral hemi-body D10 (3cm); vertebral body of D12 (2cm); and left iliac crest (1cm) were observed. ( **B**) 4 months later, a PET-TAC showed a metabolic stabilization of the disease, but with a slight growth of lesions according to MRI. ( **C**) During the following 11 months, the patient remained stable without disease progression. ( **D**) In May 2016, a 18 FDG-PET-TAC revealed an important metastatic dissemination all over the skeleton, showing more than 40 small new lesions (<2 cm), and with scarce or null Octreoscan and ^123^I-MIBG uptake. ( **E**) In December 2016, most of lesions had disappeared according to PET-TAC. ( **F**) In July 2017, a new PET supported the previous results, showing the remaining two lesions but with maximum SUV lower. ( **G**) In October 2017, 4 months since the last dose of temozolomide, and propranolol as the only treatment (240mg/day), the disease remains controlled according to this last PET-TAC.

A 53 year old man, with personal and family history of hypertension, was diagnosed with a 4 cm tumoral mass at costovertebral D6–D7 with a 21 max SUV, during a routine check-up in March 2010. The only manifestation of disease was elevated blood pressure. He was being treated at this time with irbesartan/hydrochlorothiazide (1250mg/12.5mg, respectively). After surgery, the pathology diagnosis was of PGL with a Ki67 around 5-8%, and synaptophysin and chromogranin positive. Genetic analysis found a mutation in the *SDHA* gene, leading to a heterozygous missense **p.Ser445Leu** change, defined as pathogenic ^8^. The patient remained without treatment and symptom-free until August 2014, when a back pain appeared around the D10 region, with progressive worsening. In October, as the pain persisted, he underwent MRI and 18F-DG-PET scan and was diagnosed with several metastasis at: D6–D7, right acetabulum (3cm); vertebral hemi-body D10 (3cm); vertebral body of D12 (2cm); and left iliac crest (1cm). A scintigraphy with Indium-111 pentetreotide suggested metastatic disease of probably neuroendocrine origin. D10 lesion biopsy confirmed the diagnosis of PGL metastasis with a Ki67 around 15–20% ( Figure 1A). The patient was treated with doxazosin (8 mg/day) and bisoprolol (10 mg/day).

In November 2014, the patient started on systemic treatment with denosumab (120 mg every 28 days) and lanreotide (120 mg every 14 days). After 4 months, a PET-TAC showed a metabolic stabilization of the disease, but with a slight growth of lesions according to MRI ( Figure 1B). D6 and D10 lesions were then treated by radiotherapy (Cyberknife). To this purpose, in March/April 2015 the patient underwent SBRT with CiberKnife (24 Gy in D10, 15 Gy in D12 and 35 Gy in D6–7), and the pain disappeared. After 6 months, the D7 and D12 lesions were also irradiated and completed SBRT with CiberKnife (24 Gy in left iliac bone and 24 Gy to right acetabulum).

Response to radiotherapy was good, showing stability of lesions and decrease of max SUV. No new lesions appeared. In June 2015, a thermoablation and right acetabulum metastasis cementation was performed with good results.

At this moment, the anti-hypertensive bisoprolol was changed to propranolol(120mg/day), due to preliminary reports of propranolol’s good results in VHL tumors ^9^. During the following 11 months, the patient remained stable without disease progression ( Figure 1C). Lanreotide dose was decreased to 120 mg every 3 weeks, due to side effects. Side effects included nausea, vomits, diarrhea and hyperglycaemia.

In May 2016, metanephrines and chromogranin were raised above normal levels, as follows: Chromogranin A, 106 ng/mL (normal level, <93); urine fractionated Norepinephrine, 196 mcg/24h (normal levels, 15–80); urine fractionated normetanephrine, 1316 mcg/24 h (normal levels, 128-484); urine total metanephephrine, 1394 mcg/24h (normal levels, 220-680). A 18FDG-PET-TAC revealed an important metastatic dissemination all over the skeleton, showing more than 40 small new lesions less than 2 cm, and with scarce or null Octreoscan and ^123^I-MIBG uptake ( Figure 1D).

From this moment on, a combined treatment of temozolomide (75 mg/m² for 21 days every 28 days) and an increase of propranolol up to 3 mg/kg/day (240 mg/day) was prescribed. After the first cycle of temozolomide, results were evaluated in August 2016 at National Institutes of Health, where the previously detected metastatic dissemination was confirmed, although catecholamines and chromogranin levels were decreasing compared with the previous measures. Treatment was kept the same for 6 cycles of temozolomide. In December 2016, most of lesions had disappeared according to PET-TAC ( Figure 1E). The two remaining lesions had maximum SUV significantly decreased. In January 2017, lanreotide was retired due to adverse side effects (as before).

In July 2017, a new PET supported the previous results, showing the remaining two lesions but with maximum SUV lower ( Figure 1F). Temozolomide was withdrawn because of side effects (lymphopenia and thrombopenia). In October 2017, 4 months since the last dose of temozolomide, and propranolol as the only treatment (240mg/day), the disease remains controlled according to the last PET-TAC (F18-FDG) ( Figure 1G). This imaging study suggests the presence of two neoplastic bone lesions in the dorsal spine and right acetabulum; regarding the previous examination (July 2017), the lesions described in D6 and acetabulum persist without major changes, whereas that of vertebra D10 has disappeared.

## Discussion

The present case report describes an impressive clinical and metabolic improvement of a patient suffering from a metastatic paraganglioma after using a combination of temozolomide and propranolol at high doses. The improvement has been kept even after 7 months of lanreotide removal (usually used as standard treatment).

We can only hypothesize about the reason for this spectacular result. However, there are some literature reports on the synergy between chemotherapeutic agents inducing DNA damage, and therefore blocking DNA replication (as it is here the case of temozolomide) and β-blockers, which have shown antiangiogenic and pro-apoptotic effects in tumoral cells ^9, 10^.

Recent examples of these combinations found in the literature include the action of β-blockers increasing response to different chemotherapeutic agents. Pasquier *et al.* used a mouse model of neuroblastoma, where β-blockers were shown to increase treatment efficacy ^11^. Propranolol in combination with vinblastine (chemotherapeutic agent belonging to the group of vegetable alkaloids), proved to be a successful treatment for advanced angiosarcoma ^12^, with optimal results in seven patients following a clinical trial ^13^.Chow *et al*. showed the regression of a rapidly enlarging stage T2 angiosarcoma of the scalp and face, following combination therapy with propranolol hydrochloride, paclitaxel (other vegetable alkaloid), and radiotherapy ^14^. More recently, a prospective study using propranolol for off-label treatment of patients with melanoma, confirmed a significantly inversely association with recurrence of melanoma when propranolol was used at the time of diagnosis ^15^. We could infer that propranolol seems to potentiate the anti-angiogenic effects and antitumor efficacy of chemotherapy.

Focusing our attention to propranolol- an unspecific β-blocker - as an antitumoral agent, Albiñana and coworkers ^16^ recently published the clinical and biomarker follow-up of a clinical trial conducted in seven VHL patients with retinal hemangioblastomas. These patients had no surgical option, and the tumors remained stable in size (with reabsorption of exudates) following 120 mg propranolol treatment for 12 months. This clinical trial led recently to the European Medicines Agency’s designation of propranolol as Orphan drug for VHL disease.

The hypothesis supporting the antiangiogenic properties of propranolol relies on its proven effect on decreasing HIF-inducible transcription targets ^9^. Propranolol would decrease HIF protein levels, and therefore, the activation of the hypoxia program developed by the hypoxia target genes, among them, angiogenic factors, such as VEGF, FGF, PDGF, EPO, and metalloproteases, activated in tumors that favor migration and dissemination of cancer cells.

The advantages of the use of propranolol and temozolomide derive mainly from the long experience as therapeutic agents. Both are old drugs, and therefore, the safety profile and side effects are well known. In this context, we can state that if they are proven effective in tumoral cases difficult to manage, the therapeutic solution may be immediate from the bench to the patient. In our case, after 4 months taking propranolol as the only medication, the improvement is maintained, which would support the use of propranolol as a drug that significantly increases the progression-free survival in absence of temozolomide. If these results are confirmed in more studies, its use would represent an advantage, allowing - once the disease is in remission - periods of time for the marrow to recover from the adverse effects of chemotherapy with the alkylating agent.

 The strengths of the treatment are the results and the absence of serious side effects for the propranolol in this particular case. The limitations of using propranolol derive from the anti-hypertensive effects. Hypotensive patients should take care with the treatment, and be always under cardiologist supervision.

For these rare diseases where therapeutic options are scarce and with not very successful results, outcomes like the one reported here have to be seriously considered if we want to improve the quality and life expectancy for these - in most cases - young patients.

## Consent

Written informed consent for the publication of the patient’s clinical details and images was obtained from the patient.
