# Supplementary material for: Case Report: Propranolol increases the therapeutic response to temozolomide in a patient with metastatic paraganglioma
Source: F1000Res. 2017 Dec 4;6:2087. [Version 1] doi: 10.12688/f1000research.13185.1 (PMC5824325; doi:10.12688/f1000research.13185.1)
Supplement: Peer review history

# Peer review: Referee response for version 1

**DOI:** 10.5256/f1000research.14305.r30292

**Published:** 2018-02-21

**Authors:** Carlos Jara

**Affiliations:** 1 Alcorcón Foundation University Hospital, King Juan Carlos University (URJC), Madrid, Spain

I suggest to consider this work for indexing.

 I think the subject is interesting, the presentation is appropriate and the arguments in favor of the advantage provided by propranolol are consistent. The mechanism by which this drug adds therapeutic advantage in this type of tumor remains an enigma, but I think that as a clinical case the arguments presented are sufficient.

 The description of the case is correct and detailed. The clinical evolution is surprising and the role that propranolol can exercise together with temozolamide is promising and frankly novel. The clinical improvement and its maintenance over time are striking, particularly after the failure to previous standard therapies.

 An especially positive feature of this study is to focus on a relatively rare pathology and in which the therapeutic possibilities are limited.

 For a definitive evaluation of the role that propranolol may have in the treatment of these neoplasms, together with chemotherapy, it will be necessary to develop a multicenter phase III study, which, judging by the data provided by the authors, seems to be appropriate. It is of course necessary to investigate the mechanism by which propranolol potentiate the anti-angiogenic effects and antitumor efficacy of chemotherapy.

 Suggestions for the authors:

- At the beginning the phrase "Hereditary paraganglioma and pheochromocytoma syndromes (HPP) are characterized by rare tumors called pheochromocytomas (PHEOs) and paragangliomas (PGLs)." Should be reversed by "Hereditary paraganglioma and pheochromocytoma syndromes (HPP) are characterized by rare tumors called paragangliomas (PGLs) and pheochromocytomas (PHEOs).
- ​​​​In the description of the case it should be mentioned that the study carried out presumably was an 8F-DG-PET (no data is provided other than the SUV value).
- I would suggest modifying "National Institutes of Health" by its name in Spanish, mentioning the specific hospital center.
- I would suggest modifying the expression 'spectacular' by a more restrained one as 'striking'.

I have read this submission. I believe that I have an appropriate level of expertise to confirm that it is of an acceptable scientific standard.

# Peer review: Referee response for version 1

**DOI:** 10.5256/f1000research.14305.r30293

**Published:** 2018-02-12

**Authors:** Angeles Juarranz

**Affiliations:** 1 Department of Biology, Autonomous University of Madrid, Madrid, Spain

The present case report describes an impressive clinical improvement of a patient suffering from a metastatic paraganglioma. The case is well written and documented with images. The good news is that the treatment is a combination of temozolomide and propranolol at high doses, old drugs from which the safety profile and side effects are well known.

 It is noteworthy that the improvement was kept even after 7 months of lanreotide removal. Lanreotide remains one of the choice drugs as standard treatment for metastatic paragangliome, although with high side effects, and only limited success.

 While temozolomide is commonly used for brain tumors, the novelty is the concomitant treatment with a beta blocker, propranolol. The authors explain the results based on the antiangiogenic properties of propranolol, decreasing HIF-inducible transcription targets according to previous papers published elsewhere. Propranolol would decrease HIF protein levels, and thus the activation of hypoxia target genes, among them, angiogenic factors, such as VEGF, FGF, PDGF, EPO, and metalloproteases.

 The advantages of the use of propranolol and temozolomide derive mainly from the long experience as therapeutic agents. Both are old drugs, and therefore, the safety profile and side effects are well known. In this context, we can state that if they are proven effective in tumoral cases difficult to manage.

 For rare diseases where therapeutic options are scarce and with not very successful results, outcomes like the one reported here have to be seriously considered and spread in literature, since patients may get an enormous benefit. On top of this, the proposed treatment is cheap, and with minimal side effects.

 However, this is a case report, and to be sure that the treatment really is effective, we need more knowledge derived from other cases.

I have read this submission. I believe that I have an appropriate level of expertise to confirm that it is of an acceptable scientific standard.
